# Medical students on long-term rural clinical placements and their perceptions of urban and rural internships: a qualitative study

**DOI:** 10.1186/s12909-020-02103-7

**Published:** 2020-06-10

**Authors:** Jannine Bailey, Sabrina Pit

**Affiliations:** 1 Bathurst Rural Clinical School, Western Sydney University, PO Box 9008, Bathurst, NSW 2795 Australia; 2 University Centre for Rural Health, Western Sydney University, PO Box 3074, Lismore, NSW 2480 Australia

**Keywords:** Workforce, Rural health, Intern, Perspectives, Workforce planning, Supervision

## Abstract

**Background:**

There is some anecdotal evidence that anxiety about the responsibility of an intern influences rural future intentions. Additionally, research has shown that urban interns have reported that they are worried about being ‘forced’ to work in non-metropolitan hospitals in their first year after graduation. This study sought to explore rural medical students’ perceptions and expectations of a rural internship and how local health services and/or their medical school can prepare them best for a rural intern position.

**Methods:**

Four focus groups were conducted with 62 final-year medical students upon completion of a 12-month rural clinical school placement. Focus groups were audio-recorded and transcribed verbatim for thematic analysis to identify key themes.

**Results:**

Most students have high levels of anxiety around starting work but they acknowledge that this may be exaggerated. They believe that in rural areas they get higher quality supervisory support than in urban hospitals as people know you better, whereas in the city you are more anonymous. However, the level of responsibility placed on rural interns was considered to be a double-edged sword. While rural interns were allowed to do more than be a ‘paper-pusher’ this level of responsibility means they are more accountable. The majority felt that doing your first training years in a metropolitan hospital can be crucial to getting on a training program in your chosen speciality.

**Conclusions:**

There appears to be a relatively high level of anxiety about rural internships amongst final-year medical students. Students need more targeted information around specialisation, particularly around regional training hubs, if we want to achieve higher levels of interns choosing a rural career path.

## Background

To meet the needs of rural Australians, an increase in the rural medical workforce is required [1]. One strategy of the Australian Government for attracting rural health professionals is funding rural clinical schools (RCS). A 2018 systematic review of characteristics and outcomes of Australia’s undergraduate medical rural placement programs found that programs are moderately associated with an increased supply of early career doctors although an equal number of urban-trained students contribute to rural workforce capacity [2]. Further evidence shows that rural training, experiencing the rural lifestyle and socializing increases the likelihood of students wanting to return to a rural area to practice [3, 4]. Other positive influencers include having a rural background [2, 5, 6], long-term program placements [2, 7] and early exposure to rural practice [5, 6]. McGirr and co-workers [8] found that across 10 Australian RCSs, students with a longer RCS placement were more likely to be in rural practice, after adjusting for rural background.

There is anecdotal evidence that anxiety amongst medical students about the responsibility placed on rural interns influences rural future intention. Empirical evidence is currently lacking, however, research involving urban first year graduates noted that there is a lower level of support provided in non-metropolitan placements and their anxiety is further fuelled by lack of communication through short notice of practice location and clinical placement expectations [9]. The authors concluded that “adequate professional support and supervision in rural placements” is vital to promoting rural medicine [9].

The transition from medical student to intern is a stressful time with anxiety and burnout an all too common occurrence [10, 11]. Personal experiences impact confidence levels and professional identity [10, 12]. What has not yet been elucidated are the attitudes and perceptions of final year long-stay rural medical students towards rural internships, despite the primary focus of RCSs being to build a future rural health workforce. Therefore, this study aimed to explore RCS students’ perceptions and expectations of a rural internship as compared to urban internships. A second aim was to explore how local health services and/or their medical school can prepare them best for a rural intern position.

## Methods

The study design was a qualitative study using focus groups, which covered three main topics: living/working in rural settings at all career stages, technology, and rural internships. This paper reports on rural internships.

### Participants and recruitment

Participants comprised final year medical students who had completed a year-long placement in two RCSs in New South Wales, Australia in 2017–2019. The RCSs are located in regions considered to be ‘large rural towns’ according to the Modified Monash Model criteria for classification widely used in Australia. The hospitals also service patients from outlying areas that fall into the categories of ‘medium rural towns’ and ‘small rural towns’. Prior to attending the RCS, all students had spent the preceding 18 months completing clinical placements in urban hospitals. All students (*n* = 68) were invited to partake via an email from student-coordinators. Four focus groups were conducted with 62 medical students.

### Data collection

The discussion guide utilised semi-structured open-ended questions and was developed from the literature and consultation with medical education staff at the RCSs. Table 1 displays the interview questions.

**Table 1:** Interview questions relating to a rural internship

Imagine you would work as an intern in a rural area:	
1. What would be the benefits/downsides of urban and rural internship placements?	
2. What do you think a rural internship would be like?	
3. What are your expectations in terms of level of professional and supervisory support?	
4. Can you explain why you think it is <use their words regarding their expectations for professional and supervisory support, e.g. good/bad>	
5. How can health services and/or your medical school prepare you best for a rural internship?	

Focus groups were conducted by Author2 and two research-assistants in April/May 2018/2019. The interactive focus group discussion allowed participants to show their concurrence or disagreement with the responses of others whilst also allowing them to build upon each other’s responses, resulting in the generation of data that might not have been produced in multiple individual interviews [13]. Focus groups lasted 70–90 min, were audio-recorded and transcribed verbatim. The transcripts and findings were not confirmed with the participants after the focus groups but were confirmed during the discussion. The facilitators build rapport with the students to elicit honest responses and where appropriate restated or summarised their answers, prompted them for more detail and asked them for clarification if needed to determine accuracy.

### Data analyses

Inductive thematic analysis was applied [14]. First, Author1 and Author2 read the transcripts to identify commonalities and differences. Author1 developed a draft codebook that was adapted and refined by Author2 following discussion between the authors. Themes were then identified by Author1 and refined by Author2 and consensus reached. It is unknown whether data saturation was achieved.

### Ethics

Ethics approval was granted by Western Sydney University Human Research Ethics Committee (No: H9989).

## Results

The main themes were the students’ perspectives of a rural internship contrasted with urban internships and their perceived barriers and recommendations to taking up a rural internship. The key subthemes that emerged regarding students’ perceptions of a rural internship as compared to an urban internship are summarised in Table 2. Table 3 summarises the key subthemes regarding barriers to taking up a rural internship whilst recommendations are summarised in Table 4.

**Table 2:** Key subthemes that emerged from rural undergraduate medical students (*n* = 62) regarding their perceptions of rural internships contrasted with urban internships

	Rural	Urban
**The internship role**	▪ Practical ‘hands on’ role ▪ High level of responsibility ▪ Smaller team ▪ Visibility, cannot hide and acknowledgement ▪ Work-life balance encouraged ▪ Positive culture	▪ Secretarial role; less ‘hands on’ ▪ Low level of responsibility ▪ Larger team ▪ Do not stand out
**The internship training**	▪ Generalised training experience; less variety in presentations ▪ Viable for those interested in general practice or rural generalist careers ▪ Limited training spots ▪ Uncertainty and limited understanding of training options for specialties ▪ Rural clinicians go out of their way to assist in creating rural pathways	▪ Specialised training experience; more variety in presentations ▪ Viable for those interested in all specialties ▪ Research exposure ▪ More training positions ▪ Networking required and easier to get on training programs
**Interns’ colleagues**	▪ Closer relationship with seniors ▪ Seniors more approachable/available ▪ Fewer colleagues to debrief with	▪ Less of a relationship with seniors ▪ Seniors less approachable/available ▪ Team support
**The community**	▪ Community connection ▪ Closer relationship with patients	▪ Less of a relationship with patients
**Interns’** **personal life**	▪ Social isolation	▪ Proximity to family & friends

**Table 3:** Key subthemes that emerged from rural undergraduate medical students (n = 62) regarding their perceptions of the barriers to undertaking a rural internship

	Barriers to undertaking a rural internship
**Lack of knowledge of rural training**	▪ Limited information provided by the medical school, rural clinical school and local hospitals about rural internships
**Internship training**	▪ Limited exposure to rarer or higher acuity presentations ▪ Limited research opportunities, particularly lab-based research
**Personal factors**	▪ Relocation ▪ Loss of existing family & friend support networks ▪ Fear of the unknown
**Future considerations**	▪ Limited rural training pipeline ▪ Fear of missing out on the networking opportunities available in urban; this would disadvantage them when applying for specialty training

**Table 4:**
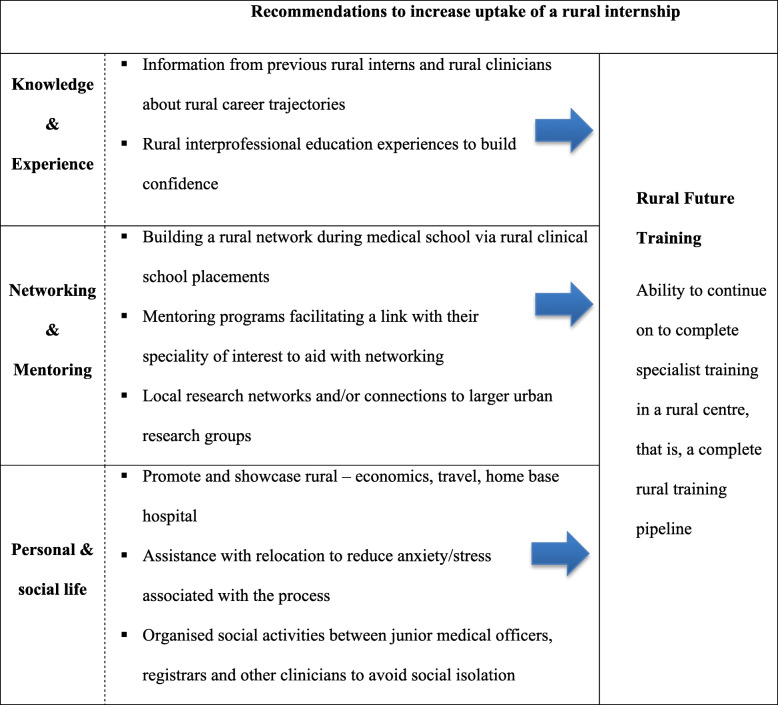
Key recommendations that emerged from rural undergraduate medical students (n = 62) that might increase uptake of a rural internship

### Student perceptions of rural versus urban internships

Students expressed many positive perceptions of rural internships, but did acknowledge some drawbacks (Table 2). Key subthemes were: their perceptions of the rural internship role, rural internship training, rural colleagues, rural communities, and their personal life in a rural setting.

A rural internship provides a more practical and clinical ‘hands-on’ opportunity compared to urban internships which were viewed as being less ‘hands-on’ and tending towards being a secretary role. This increased practical role in a rural setting leads to a higher level of responsibility. A rural intern in a smaller team was perceived as being more visible and receiving more acknowledgement for the work they did; but this also meant that they could not hide and were more accountable, both to their seniors and to the community. Students felt this would also increase their anxiety. As an urban intern they felt that they would not stand out and would blend into the much larger team, but this meant that they would not necessarily be acknowledged for their work.

*“I feel like the acknowledgement of the work that you do is I think easier to see and easier to do when you’re in rural because it’s smaller, but that being said, you are accountable even more so, if that makes sense.”*

*“Something I’ve seen with the juniors here, I think they get a lot more out of the terms, but often they are under a bit more pressure or they’ve got to do a little bit more, which is a great way to be a better doctor, but for some people it would be stressful, and that may be a downside.”*

Students viewed rural health services as actively promoting a good work-life balance amongst their staff, including junior doctors. They felt that their well-being was as much of a focus as their rural training experience. They also perceived that a more ‘positive culture’ exists in rural health services. However, in terms of their personal life, they also viewed social isolation as a potential downside, particularly when having to relocate away from existing support networks such as family and friends. This was counterbalanced in that rural community networks are considered strong.

Rural internships were viewed as providing a more generalised training experience with less variety in presentations, particularly high acuity presentations, and limited exposure to rare cases. Such training was viewed positively compared to specialised training provided in urban internships. However, they also felt that the greater variety and higher acuity presentations they would be exposed to in urban hospitals were of benefit. Rural internships were viewed as beneficial for those interested in general practice or rural generalist pathways but not for other specialties. Uncertainty and limited understanding of rural training options arose. Some students noted that rural clinicians go out of their way to assist interns in working towards rural speciality training pathways, something they had not witnessed in urban hospitals. Overall students felt that it was easier to get on urban training programs. The limited training positions in rural compared to urban was also mentioned.

Many felt that rural interns have a closer relationship with senior colleagues than urban interns. Rural senior colleagues were seen as being more approachable and available. Students’ also appreciated the community connection and closer patient relationship in rural settings. However, they also felt they would have more team support during urban internships with fewer colleagues to debrief with in rural settings.

### Barriers to a rural internship

Students identified various barriers to rural internships based on knowledge, training experience, personal factors and future considerations (Table 3).

Students felt that their RCS, the broader medical school and the local hospitals did not provide them with sufficient information about opportunities for rural internships, that is, they did not sell the opportunity to them.

Fear of missing out on developing their skills with higher acuity presentations and rarer cases was a clear barrier. Students thought these cases were immediately transferred to the urban centres. For some, the limited opportunity to conduct lab-based research was also a barrier.

*“I know some people are also concerned about the lack of research opportunities rurally. So in terms of career progression, getting those lines on your resume, that sort of stuff.”*

On a personal level, having to relocate their whole life and leaving behind support networks was a barrier. These networks were seen as a vital component to maintaining their well-being during the stressful intern year. Students felt that fear of the unknown would be a major barrier for many of their urban-based colleagues in taking up a rural internship position.

Looking towards future needs, students perceived a lack of regional training places, or incomplete training pipelines, for many specialties and the need to then relocate back to an urban region if they wished to train in these specialties as barriers.

*“I could see myself working in a more regional area. But for any of the specialties that I could see myself doing, the start of the training program for all of those is in a metropolitan area.”*

As a perceived ‘outsider’ when applying for a specialty training position in urban hospitals, students felt that they would be overlooked in favour of insiders already working there. Additionally, a fear existed of missing out on making valuable urban connections/networks. If they were a rural intern, they would not be able to establish these networks and this would disadvantage them when applying for speciality training.

*“I think in our mind ... is that like if we go to a city hospital, … , we might be able to make connections with consultants and with the kind of teams that will let us go into pathways like basic physician training and that kind of thing. I guess there’s a fear that if we go to the country no one will know who we are if we then come back later. It might be completely unfounded but that is where we kind of think. So that makes it a big part of our decision.”*

*“Lots of doctors and registrars and residents will say it’s a bit hard to crack into new hospitals or the big city hospitals as an outsider, and so if you do your first couple of years out in the country it’s hard to go back for a lot of things.”*

### Recommendations to increase rural internship uptake

Students identified various recommendations that might increase rural internships uptake, based on knowledge and experience, networking/mentoring, their personal and social life. These themes were all linked to a theme rural future training (Table 4).

To break down any potential misconceptions and reduce potential anxiety about ‘going rural’, students recommended increasing their knowledge of rural internships and experience. Information sessions where rural interns talk about their experiences and clinicians talk about rural career trajectories would be beneficial.

*“But if they could just provide a bit more guidance about what career trajectories can happen rurally, I think that would really help people and encourage them to stay rurally as well.”*

Reducing internship anxiety through communication training and assertive leadership models was mentioned by students as a means of promoting their confidence with being led in a team during their rural internship and their confidence in communicating effectively with patients and staff. Facilitating rural interprofessional education experiences at medical school was also recommended for confidence building. Students suggested that rural networks could be built during their medical training, inclusive of all RCS students. Mentoring programs that facilitate links with urban clinicians in their specialty of interest whilst they are interning in a rural setting would aid with networking for future applications to specialty training. The existence of local research networks and/or connections to larger urban research groups would provide important research opportunities.

Showcasing rural life would also be a drawcard. Students specifically mentioned the lower cost of living in rural areas (e.g. rent) and the reduced and/or easier travel to work. In some rural training posts, interns are solely based at one hospital and are not required to rotate to other hospitals. Students felt that this was a benefit worth promoting as for many urban internships there was a need to rotate between hospitals and this has a substantial impact on their travel time.

*“Less stress in terms of traffic, less stress in terms of that renting. Also less stress in terms of you’re placed at one hospital for the whole time as opposed to having to rotate between hospitals and traffic in the city.”*

*“Financially I think it would be better here, because you’re probably getting paid the same amount as if you lived in Sydney or a city, but the cost of living is cheaper.”*

To further support them on a personal level, they suggested the rural health service could provide them with relocation assistance to reduce some of their anxiety around this, such as advice and support with housing and travel arrangements. On a social level, they recommended organised social activities between junior medical officers, registrars and other clinicians to help avoid social isolation and promote well-being.

*“Sorting out all the peripheral stuff, like making sure your housing and travel and things like that are all sorted so that the stress outside of the medical placement is at a minimum. Because coming out here is particularly challenging.”*

Students identified a major recommendation to undertaking a rural internship would be the presence of a complete rural training pipeline. This alone would counter many of the barriers to ‘going rural’ as they would not be required to return to an urban setting to complete their specialty training and they could network locally to identify mentors to support them in their rural career trajectory.

## Discussion

This study explored rural medical students’ perceptions and expectations of a rural internship and how local health services and/or their medical school can prepare them best for a rural internship. Overall, students reported positive perceptions of rural internships, including a higher level of quality supervisory support, a closer relationship with senior colleagues, and having higher levels of responsibility due to higher clinical or ‘hands-on’ workloads when compared to urban internships. However, this was considered a double-edged sword as being more than just a ‘paper pusher’ makes them more accountable and visible without a larger team to hide behind. This finding is confirmed by recent research which interviewed urban junior doctors about their experiences of making the transition from medical student to intern [10]. They reported feeling unprepared for the administrative workload required of them as a junior doctor, which took up most of their day. Bonney and colleagues [15] found that rural junior doctors saw more patients per day than urban junior doctors. They also found that both rural and urban junior doctors spoke highly of their experiences and being placed in a rural hospital was not viewed as being a disadvantage [15]. In fact, rural junior doctors perceived they had more exposure to higher acuity presentations than their urban counterparts who reported that these were dealt with by registrars. Rural junior doctors also reported greater accessibility to senior colleagues and this facilitated strong and positive relationships [15], a finding which aligns with our findings.

Students identified a lack of knowledge of rural training, the internship training experience, personal factors and future considerations as key barriers to rural internships. They recommended the creation of a rural training pipeline or rural training centre. This was linked to improving their rural knowledge and experience, rural networking and mentoring, and support their personal/social life to settle in a rural area. This recommendation is supported by research among early career clinicians that showed that positive rural placement experiences were linked to quality supervision and professional support [9]. Other recently funded initiatives such as the Federal Government’s Regional Training Hubs [16] and the Rural Doctors Network’s Rural Health Pro [17] may address some of the recommendations for facilitating rural internship uptake. Regional Training Hubs aim to support the expansion of existing rural training pipelines so that there are opportunities for trainees to complete as much of their training rurally as is possible [16]. Rural Health Pro on the other hand is being implemented to provide a virtual networking forum for rural health professionals to mitigate feelings of professional isolation [17]. A pipeline of specialty training options, including but not limited to general practice and rural generalism pathways, along with a health professional support network would alleviate student concerns.

### Strengths and limitations

Only four focus groups were conducted with relatively large groups, however, almost all final year medical students participated. It is unclear whether data saturation was reached, but recent research identified that most themes emerge during the 1st focus group (> 60%) or second (> 73%) [18]. ‘Speed of data saturation’ is more likely to be achieved when 1) using more structured interview questions, as in our case; and 2) with a more homogeneous participant group, i.e. final year RCS medical students who all experienced a year living rurally [19]. The results may not be generalizable to other RCS. Future research should seek to include the rural internship perspectives of wholly urban based medical students as a comparator against those students who complete a long-term RCS placement.

### Implications and recommendations

Medical students’ current knowledge and perceptions influence rural intern uptake. Following the students’ recommendations may increase uptake. We recommend that regional training hubs work collaboratively with students to develop programs that align with students’ preferences. Future research could focus on investigating the development of regional training hubs and the perceptions of key stakeholders. Future workforce planning policy may look into establishing long-term rural training pathways. This could increase workforce capability in rural and remote areas.

## Conclusion

Promotion and student education around rural internships and rural postgraduate pathways is needed to increase rural intern uptake. Students are aware of some of the potential benefits of working rurally as a junior doctor but these are overshadowed by a ‘fear of missing out’ both in terms of clinical exposure on the ward and professionally in terms of creating networks to support them with entering specialty training pathways. Workforce planners, clinicians, policy makers and educators should develop strategies to increase the uptake of rural internship and development of long-term rural training pathways to improve workforce capability in rural Australia.

## Data Availability

The datasets are not available from the corresponding author due to the sensitive nature of the data and the consent being provided for participation in the specific study.

## Abbreviation

RCS: Rural Clinical School
